# Cumulative incidence of post‐infection asthma or wheezing among young children clinically diagnosed with respiratory syncytial virus infection in the United States: A retrospective database analysis

**DOI:** 10.1111/irv.12770

**Published:** 2020-06-12

**Authors:** Jonathan Nguyen‐Van‐Tam, Veronique Wyffels, Maartje Smulders, Debasish Mazumder, Rohit Tyagi, Nikhil Gupta, Sandra Gavart, Roman Fleischhackl

**Affiliations:** ^1^ Health Protection and Influenza Research Group University of Nottingham Nottingham UK; ^2^ Janssen Pharmaceutica NV Beerse Belgium; ^3^ SmartAnalyst New York City NY USA; ^4^ SmartAnalyst Gurgaon India; ^5^ Janssen‐Cilag Vienna Austria

**Keywords:** asthma, cumulative incidence, infants, pre‐existing high‐risk factors, respiratory syncytial virus, wheezing

## Abstract

**Background:**

Respiratory syncytial virus (RSV) infection is implicated in subsequent development of asthma/wheezing (AW) among term and pre‐term infants. We describe the cumulative incidence of AW among hospitalized and ambulatory neonates/infants/toddlers following RSV infection diagnosis over three independent follow‐up periods.

**Methods:**

Between January 1, 2007 and March 31, 2016, patients aged 0‐2 years old with first clinical diagnosis of RSV infection were identified using the Optum^®^ integrated electronic health records and claims database. Patients diagnosed with AW ≤ 30 days post‐RSV diagnosis were excluded. Three cohorts with 1, 3, and 5 years of follow‐up were stratified by presence or absence of specific RSV high‐risk factors, including pre‐term birth and pre‐defined, pre‐existing comorbidities. Descriptive statistics and logistic regression results were reported.

**Results:**

Overall, 9811, 4524, and 1788 RSV‐infected high‐risk factor negative patients were included in 1, 3, and 5‐year independent cohorts, respectively. Of these, 6.5%, 6.9%, and 5.8%, respectively had RSV‐related hospitalization. By the end of follow‐up, 14.9%, 28.2%, and 36.3% had AW events. Overall, 3030, 1378, and 552 RSV‐infected high‐risk factor positive patients were included in the respective cohorts. Of these, 11.4%, 11.1%, and 11.6%, respectively were hospitalized with initial RSV infection and 18.1%, 32.9%, and 37.9% had subsequent AW events within the follow‐up period. Logistic regression confirmed RSV‐related hospitalization significantly increased the likelihood of developing AW (*P* < .05) in high‐risk factor positive and negative patients.

**Conclusions:**

In infants diagnosed with RSV infection, RSV‐related hospitalization was associated with a significantly increased likelihood of AW development for at least 5 years, compared with non‐hospitalized patients.

## 1 INTRODUCTION

Acute lower respiratory tract infections (LRTIs) caused by respiratory syncytial virus (RSV) infection are a common cause of hospitalization in the young, especially in children under 2 years of age. Globally in 2015, approximately 3.2 million (uncertainty range [UR] 2.7‐3.8) hospital admissions, and 59 600 (UR 48 000‐74 500) in‐hospital deaths occurred worldwide due to RSV‐related LRTIs in children younger than 5 years old. In children younger than 6 months, 1.4 million (UR 1.2‐1.7) hospital admissions and 27 300 (UR 20 700‐36 200) in‐hospital deaths were recorded due to RSV LRTI.^1^ Studies indicate that among children, RSV is the causative pathogen of 50%‐90% of hospitalizations due to bronchiolitis, 5%‐40% of those due to pneumonia, and 10%‐30% of those due to tracheobronchitis.^2^, ^3^ In the USA, RSV infection has been associated with an estimated 57 527 hospitalizations and 2.1 million outpatient visits each year among children aged < 5 years.^4^

Although RSV infection is self‐limiting in most patients, severe outcomes and complicated disease are more likely in certain populations, for example, infants under 6 months of age, pre‐term infants, older adults (≥65 years old), immunocompromised patients, and patients with chronic lung or heart disease.^5^, ^6^, ^7^ Mortality rates associated with RSV infection are generally low in otherwise healthy infants (below 1%), but increase significantly in children with bronchopulmonary dysplasia (BPD) and congenital heart disease (CHD).^7^ A previous study reported a threefold higher mortality rate in children with cardiac (3.4%) and lung disease (3.5%).^8^ Another study reported the weighted mean case fatality rate was 1.2% among pre‐term infants; 5.2% among children with CHD; and 4.1% among children with BPD. Case fatality estimates among children not at high risk ranged from 0% to 1.5%.^9^

Although wheeze is a key symptom of RSV infection itself, further episodes of acute wheezing are reported following RSV LRTI at an early age, particularly in the first 2 years of life. These episodes mimic early childhood asthma, with persistence of lung function abnormalities until adolescence in some patients. This phenomenon has been termed post‐RSV wheezing disorder.^7^, ^10^ Some evidence also exists that children contracting RSV bronchiolitis within the first year of life may be at increased risk of developing asthma later in childhood.^11^ Studies have demonstrated that children hospitalized with RSV bronchiolitis during infancy are more likely to have subsequent episodes of wheezing and asthma compared with children with RSV bronchiolitis but not hospitalized, particularly in the first 4 years of life.^12^

At present, understanding of the post‐RSV infection clinical burden of disease in infants and young children, both with and without pre‐existing high‐risk factors, remains limited. Most recent studies have focused on premature infants at high risk for asthma/recurrent wheezing. In this study, we describe the cumulative incidence of post‐RSV infection asthma/wheezing (AW) among a large general population of children aged 0‐2 years (including both high‐risk factor positive and negative children), after a clinical diagnosis of RSV bronchiolitis using an integrated EHR and claims database.

## 2 METHODS

### 2.1 Study aims and design

We aimed to calculate the cumulative incidence of AW among neonates, infants, and toddlers (aged 0‐2 years) after RSV diagnosis over separate follow‐up periods of 1, 3, or 5 years, using an Integrated EHR and claims database. These 3 separate follow‐up periods were established to compensate for data lost to follow‐up and to accurately reflect the true post‐RSV infection AW cumulative incidence rates in the target population. We explored the a priori hypothesis that the cumulative incidence of post‐RSV infection AW would be different between independent groups of patients: those hospitalized with RSV infection vs those managed exclusively via ambulatory care.

### 2.2 Case records and methodology

This study used integrated de‐identified claims and EHR data from the Optum Integrated EHR and claims database to identify neonates/infants/toddlers (0‐2 years old) diagnosed with RSV infection/unspecified bronchiolitis between January 1, 2007 and March 31, 2016. Clinical diagnosis of RSV infection/unspecified bronchiolitis was confirmed based on ICD‐9 diagnosis codes (including 480.1, 79.6, 466.11, and 466.19) and ICD‐10 diagnosis codes (including J12.1, B97.4, J21.0, J20.5, J21.8, and J21.9). Diagnosis codes for AW included ICD‐9 code 493 and ICD‐10 codes J45 and R06.2, with only the first ever AW diagnosis considered for this analysis (Table S1). The database holds records for over 2.2 million people in the USA and is broadly representative of the US population as a whole. However, it contains relatively more data from the Mid‐West and less from the West and North‐East regions, while the proportion of its data from the South region is similar to the national geographical distribution. The Optum database has a lower percentage of younger patients (7% aged 0‐9 years) compared with the US population as a whole (13% aged 0‐9 years).

### 2.3 Study population

**FIGURE 1:**
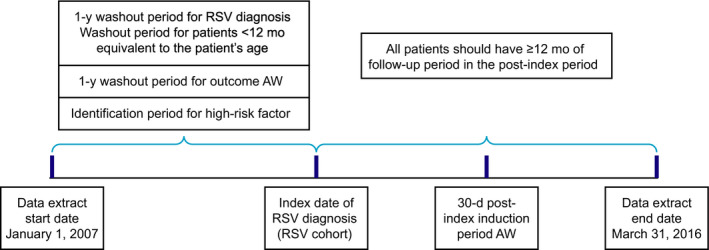
Study design diagram. AW, asthma/wheezing; RSV, respiratory syncytial virus

Patients with a clinically recognized (ICD‐9 or ICD‐10 recorded) RSV infection diagnosis during hospitalization, emergency room, ambulatory, or hospital outpatient visits were identified in the database. All patients were required to have at least 1 year of data prior to diagnosis (Figure 1). In patients < 12 months of age, data before first RSV infection diagnosis were required for a period equivalent to the patient's age. Patients with an initial diagnosis of AW ≤ 30 days during or after first RSV diagnosis were excluded from the analysis (induction period).

Stratified analyses were undertaken on patients with and without a clinical diagnosis of high‐risk factors for AW. High‐risk factors included pre‐term birth, cardiovascular disease (including cerebrovascular disease, Down's syndrome with CHD, ischemic heart disease, peripheral vascular disease, higher risk/lower risk CHD), bronchopulmonary disease (asthma, BPD, pneumonia [excluding that due to RSV infection], interstitial pulmonary fibrosis of prematurity, Wilson‐Mikity syndrome, congenital airway anomalies, chronic perinatal respiratory disease), and neuromuscular disease. Other high‐risk factors included cystic fibrosis, congenital and metabolic disease, and kidney disease. Occurrence of ICD codes for high‐risk factors was identified during the 12 months preceding first RSV diagnosis for patients > 1 year of age, except pre‐term birth which was identified during or after RSV diagnosis. For patients < 1 year of age, high‐risk factor diagnosis was considered at any time during their lifetime. Following RSV diagnosis, new development of these predefined risk factors was not assessed. For full details on factors considered high risk and their diagnosis codes, see Table S1.

Hospitalizations were considered RSV‐related if the admission was associated with ICD‐9 or ICD‐10 codes for RSV infection and if the hospitalization occurred during the follow‐up period (1/3/5 years) at any time before the incidence of AW; this includes patients diagnosed in an outpatient setting who were subsequently hospitalized with an RSV diagnosis, as well as patients with an initial diagnosis of RSV in the hospital setting. Both RSV infection and respiratory plus concurrent RSV diagnosis hospital admission claims in the EHR were included. Hospital admissions without RSV diagnosis codes were excluded. Severe RSV cases are considered more likely to be treated in the hospital than ambulatory patients. For patients with multiple comorbidities, the order in which diagnoses are recorded may differ, therefore, all (primary, secondary, or tertiary) diagnosis code positions were used for identification.

### 2.4 Statistics

Frequencies (percentages with 95% confidence intervals [95% CI]) and means (standard deviation) are reported for categorical (eg, gender, and race) and continuous variables (eg, age) for cohorts with or without high‐risk factors, respectively. Logistic regression analyses were conducted to assess whether patients with a clinical diagnosis of RSV infection who were hospitalized had a significantly larger likelihood of developing post‐RSV infection AW compared with non‐hospitalized patients.

## 3 RESULTS

A total of 12 841 patients diagnosed with RSV infection had at least 1 year of follow‐up data, while a further 5902 and 2340 patients had 3 and 5 years of follow‐up data, respectively. Among these groups, 9811, 4524, and 1788 patients respectively were high‐risk factor negative and 3030, 1378, and 552 patients respectively were high‐risk factor positive. Attrition of patients throughout the study period is described in Figure S1.

**FIGURE 2:**
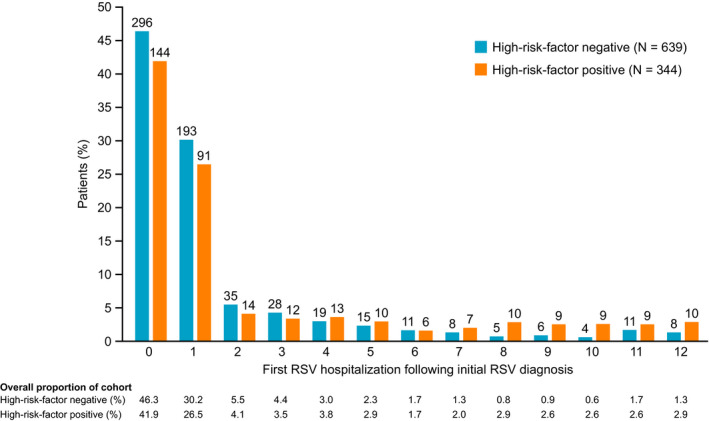
Timing of first RSV hospitalization for the cohort of patients with 1 y of follow‐up. RSV, respiratory syncytial virus

The demographic characteristics of the patients with 1 year of follow‐up are summarized in Table 1, with 56.7% males in the high‐risk factor negative patients group and 57.1% males in the high‐risk factor positive patients group, and an overall mean age of 0.8 years at first RSV diagnosis (0.7 years for high‐risk factor negative and 0.8 years for high‐risk factor positive patients). By the end of 1 year of follow‐up, the overall proportion of patients with RSV‐related hospitalization was 7.7%, while the hospitalization rates by risk factor were 6.5% for high‐risk factor negative patients and 11.4% for high‐risk factor positive patients. The majority (>70%) of RSV‐related hospitalizations occurred within the first 2 months following initial RSV diagnosis (Figure 2). The other 2 cohorts (3 and 5 years of follow‐up) showed similar demographic patterns, with similar hospitalization rates according to the presence or absence of high‐risk factors across both follow‐up cohorts (Table S2).

**TABLE 1:** Demographic characteristics of patients with RSV infection and 1 year of follow‐up

	Overall	High‐risk factor negative (76.4%)	High‐risk factor positive (23.6%)
	All patients (n = 12 841)	All patients (n = 9811)	All patients (n = 3030)
Gender			
Male, n (%)	7292 (56.8)	5561 (56.7)	1731 (57.1)
Age^a^			
Mean (SD)	0.8 (0.9)	0.7 (0.9)	0.8 (0.9)
Median (IQR)	0 (0‐2)	0 (0‐2)	0 (0‐2)
Race			
African American, n (%)	630 (4.9)	478 (4.9)	152 (5.0)
Asian, n (%)	491 (3.8)	370 (3.8)	121 (4.0)
Caucasian, n (%)	8781 (68.4)	6683 (68.1)	2098 (69.2)
Other/unknown, n (%)	2939 (22.9)	2280 (23.2)	659 (21.8)
Hospitalized with RSV^b^, ^a^, n (%)	983 (7.7)	639 (6.5)	344 (11.4)

Abbreviations: IQR, interquartile range; RSV, respiratory syncytial virus; SD, standard deviation.

^a^ Age at first RSV and or bronchiolitis diagnosis in years.

^b^ Hospitalization during and within the respective follow‐up post first RSV diagnosis.

### 3.1 Cumulative incidence of post‐RSV infection AW

**FIGURE 3:**
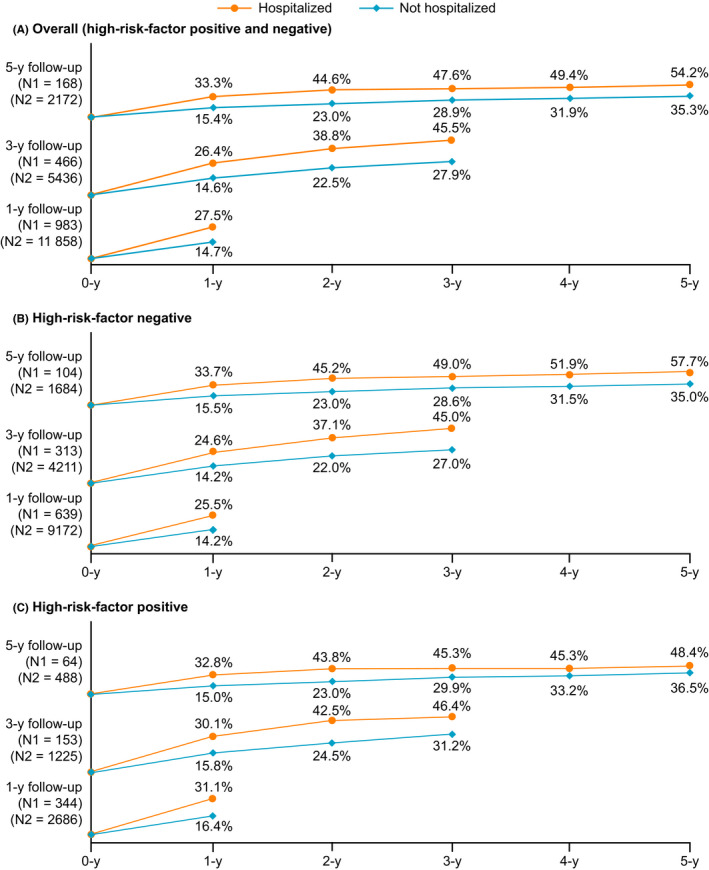
Cumulative incidence of AW among hospitalized/ambulatory neonates/infants/toddlers after RSV/bronchiolitis infection diagnosis in the Optum Integrated Electronic Health Records and claims database. AW, asthma/wheezing; RSV, respiratory syncytial virus

Among the 1‐year follow‐up cohort, 2009 of 12 841 (15.6%) patients had an AW diagnosis, including 1462 of 9811 (14.9%) who were high‐risk factor negative and 547 of 3030 (18.1%) who were high‐risk factor positive. For patients with RSV‐related hospitalization, 27.5%, 45.5%, and 54.2% by the end of the 1, 3, and 5 years of follow‐up, respectively, experienced at least 1 AW event (Figure 3A). The cumulative incidence of post‐RSV infection AW was lower among ambulatory care only patients (14.7%, 27.9%, and 35.3%, respectively) (Figure 3A) compared with patients with RSV‐related hospitalization.

A similar pattern of higher post‐RSV infection AW incidence in hospitalized vs ambulatory patients was observed among high‐risk factor negative (Figure 3B) and among high‐risk factor positive patients (Figure 3C).

### 3.2 Association between hospitalization and risk of post‐RSV infection AW

Logistic regression analysis indicated that RSV‐related hospitalization is associated with a significantly increased likelihood of developing AW among both high‐risk factor positive and high‐risk factor negative RSV‐infected patients at each follow‐up year, compared with RSV infection managed solely in ambulatory care, typically by twofold (Table 2). Males also appeared to be at consistently elevated likelihood for developing AW, typically 1.5‐fold, whereas RSV diagnosis in the known RSV season (as opposed to other times of year) was associated with a typically 30% lower likelihood of AW (Table 2).

**TABLE 2:** Odds ratios for risk of developing AW among hospitalized/ambulatory neonates/infants/toddlers after RSV/bronchiolitis infection diagnosis, in the Optum Integrated Electronic Health Records and claims database

	High‐risk factor negative			High‐risk factor positive		
	1‐y follow‐up	3‐y follow‐up	5‐y follow‐up	1‐y follow‐up	3‐y follow‐up	5‐y follow‐up
RSV hospitalization^b^, ^a^ (yes vs no)						
Odds ratio	2.1	2.3	2.7	2.4	2.0	1.7
Confidence interval	1.8‐2.6	1.8‐2.9	1.8‐4.0	1.8‐3.0	1.4‐2.8	1.0‐3.0
*P* value	<.0001	<.0001	<.0001	<.0001	<.0001	.0433
RSV diagnosis in RSV season (yes vs no)						
Odds ratio	0.7	0.7	0.6	0.7	0.7	0.8
Confidence interval	0.6‐0.8	0.6‐0.8	0.5‐0.8	0.6‐0.9	0.5‐1.0	0.5‐1.3
*P* value	<.0001	<.0001	<.0002	.0083	.0264	.3388
Gender (male vs female)						
Odds ratio	1.5	1.5	1.5	1.4	1.3	1.7
Confidence interval	1.3‐1.7	1.3‐1.7	1.2‐1.8	1.2‐1.7	1.0‐1.6	1.2‐2.5
*P* value	<.0001	<.0001	<.0002	<.0007	.0311	.0036
Caucasian (yes vs no)						
Odds ratio	0.9	0.8	0.9	0.9	0.9	1.0
Confidence interval	0.8‐1.0	0.7‐0.9	0.7‐1.1	0.7‐1.1	0.7‐1.2	0.7‐1.5
*P* value	.0069	.0049	.1949	.3836	.6169	.8096
Prior palivizumab use (yes vs no)						
Odds ratio	‐	‐	‐	1.2	1.1	1.6
Confidence interval	‐	‐	‐	0.8‐1.7	0.7‐1.7	0.8‐3.3
*P* value	‐	‐	‐	.4187	.643	.1866

Abbreviations: AW, asthma/wheezing; FU, follow‐up; RSV, respiratory syncytial virus.

^a^ Hospitalization during and within the respective follow‐up post first RSV diagnosis.

## 4 DISCUSSION

We used data from the Optum Integrated EHR and claims database to investigate the cumulative incidence of AW among a large population of hospitalized and ambulatory neonates, infants, and toddlers diagnosed with RSV infection, with and without pre‐existing high‐risk factors. Overall, we found that the cumulative incidence of AW within 5 years of diagnosis was high in both ambulatory RSV infection patients (35%) and patients hospitalized with RSV bronchiolitis (54%). Results suggest that while severity of RSV infection is a marker for AW later in childhood, and severe cases are more likely to require hospitalization, milder cases of RSV infection may still be associated with post‐RSV infection AW. After 1 year of follow‐up, in RSV‐related hospitalized patients the incidence of AW among high‐risk factor positive patients is higher than among high‐risk factor negative patients. At 5‐year follow‐up, there was a smaller numerical difference in the AW incidence between these 2 groups. This may be due to the smaller sample sizes and/or development of high‐risk factors after RSV diagnosis.

A previous study looked at asthma and wheezing in infants following hospitalization due to RSV bronchiolitis.^13^ This study reported a cumulative prevalence of asthma of 30% in the RSV group vs 3% in the control group at age 7 years (*P* < .001), while the cumulative prevalence of "any wheezing" was 68% and 34% in these groups, respectively (*P* < .001). Asthma was reported during the year prior to follow‐up in 23% of the RSV group vs 2% of the control group (*P* < .001), while allergic sensitization was found in 41% vs 22% of the control group (*P* = .039). RSV bronchiolitis had the highest independent risk ratio for asthma (odds ratio [OR]: 12.7) and had a significantly elevated independent risk ratio for allergic sensitization (OR: 2.4). In addition, a longitudinal cohort study found that in infants and children previously hospitalized with RSV‐proven bronchiolitis, the prevalence of wheezing was 28.1% vs 13.1% in controls at 30‐42 months old. At age 69‐81 months, this was 22.6% vs 9.6%, respectively. The cumulative prevalence of asthma was 38.4% in the RSV group and 20.1% in controls at 91 months old.^14^

Given the association between RSV bronchiolitis and subsequent risk for AW, it is plausible that protecting young children with vaccines or antibody therapies might also reduce the subsequent burden of wheezing disorders in childhood. Palivizumab, a monoclonal antibody, is currently licensed for prevention of RSV infection in a limited population. A recently published follow‐up of a randomized control trial analyzed the effect of palivizumab prophylaxis during infancy on asthma and lung function in children aged 6 years. While the incidence of parent‐reported current asthma was significantly decreased in the palivizumab group compared with placebo (14.1% vs 24.0%; a 9.9% absolute risk reduction), palivizumab prophylaxis had no significant effect on the incidence of current physician‐diagnosed asthma (10.3% vs 9.9%, risk ratio—0.4) or lung function.^15^

In contrast, a Japanese observational case‐control study in children aged 6 years evaluated whether palivizumab could impact subsequent recurring wheeze and atopic asthma. Physician‐diagnosed recurrent wheezing was significantly lower in treated infants compared with untreated infants (15.3% vs 31.6%; *P* = .003). However, there was no difference between treated and untreated groups in the rate of atopic asthma (15.3% and 18.2%, respectively).^16^

It is difficult to compare the contrasting findings of these studies due to differences in study designs, endpoint definitions, and ethnic profiles of participants. Taken together, these findings suggest prophylactic treatment may be effective in reducing RSV‐related morbidity in infants. However, palivizumab use is currently limited to high‐risk patient populations and as such, there is an unmet medical need for the development of simpler RSV treatments which protect the more generalized population of infants from severe RSV infection and associated complications, such as AW.

In our study, RSV diagnosis in the RSV season was associated with a typically 30% lower likelihood of AW compared with other times of year (Table 2). This may indicate that RSV diagnosis outside the known RSV season carries a higher risk of AW, possibly due to patients being more susceptible to RSV at this time—this result would require further investigation before any conclusions may be drawn.

As with any such study, our study has a number of limitations, most notably its retrospective, observational nature which means that it is not possible to infer cause and effect between hospitalization and AW risk. In addition, the study was designed to evaluate patients with a first‐time diagnosis of RSV or AW; however, it does not include recurrent AW episodes which are well recognized in other studies.^17^, ^18^ It is also not possible to determine the atopic status of the infants/children studied using Optum. The database has a lower percentage of younger patients than the US population as a whole; therefore, the study population may be under‐represented. While it is possible that RSV‐infected patients may not have been adequately captured in the database, it is reasonable to expect that any such under‐representation would be consistent across all the study cohorts.

Another issue relating to the study methodology is that RSV diagnosis was identified by the appearance of an RSV/bronchiolitis ICD‐9 or ICD‐10 code on a medical claim or record. Thus, some bronchiolitis patients may not have had RSV infection, as bronchiolitis could have been caused by another etiologic agent. Although RSV infection is the most common cause of bronchiolitis, approximately one‐third of cases can be caused by another viral pathogen.^19^, ^20^ A recent study^21^ reported that RSV‐specific ICD‐10 codes may underestimate the number of actual RSV infections and suggested that a combination of RSV‐specific and general acute LRTI ICD‐0 codes may be more accurate; therefore, our approach could possibly underestimate the true burden of RSV. Also, in Optum, a medical record does not indicate that an RSV diagnostic test was performed in that setting and diagnosis may have instead been based on symptoms, risk factors, and seasonality only. RSV laboratory test results in the database were not comprehensively recorded and therefore were not used for RSV patient identification. Additionally, as high‐risk factors were only assessed before first RSV diagnosis, the impact of any newly emerging high‐risk factors cannot be evaluated. Patients hospitalized with RSV infection may have been more likely to visit a hospital specialist than a primary care physician for any subsequent AW; hospital specialist visits are not captured in the Optum database.

However, an important strength of the use of the Optum Integrated EHR and claims database in relation to the current study is that it contains linked data on the whole patient journey, encompassing ambulatory, and hospital care.

In summary, this large population database study has provided further evidence of the incidence of AW among neonates, infants, and toddlers after RSV infection. We have identified an increased likelihood of developing AW among RSV‐infected patients who were hospitalized compared with patients managed exclusively in ambulatory care. To reduce the burden of wheezing disorders in children, efforts should be directed toward finding new vaccines to prevent RSV infection and developing antiviral therapies to prevent severe disease, early diagnosis, and interventions which reduce the risk of RSV severity.

## CONFLICT OF INTEREST

Jonathan Nguyen‐Van‐Tam declares no conflicts of interests. In particular, he has never received any form of payment from Janssen Pharmaceutica, Belgium or its contractors for his role in this or any other work. He is currently on secondment to the Department of Health and Social Care (DHSC), England. The views expressed in this manuscript are those of the authors and not necessarily those of the DHSC. Maartje Smulders, Debasish Mazumder, Nikhil Gupta, and Rohit Tyagi received research funding from Janssen Pharmaceutica, Belgium. Veronique Wyffels, Sandra Gavart, and Roman Fleischhackl are employed by Janssen Pharmaceutica, a Johnson & Johnson company and may be Johnson & Johnson stockholders.

## AUTHOR CONTRIBUTIONS

MS, DM, NG, SG and RF were involved in the in the conception and design of the study. MS, NG and RF were responsible for the data collection and acquisition. JGNVT, VW, MS, DM, RT and NG were involved in the analysis and interpretation of the data. All authors were involved in the drafting of the manuscript. The authors vouch for the accuracy and completeness of the data and analyses.

## Supporting information

Supplementary Material
