# Supplementary material for: Cumulative incidence of post‐infection asthma or wheezing among young children clinically diagnosed with respiratory syncytial virus infection in the United States: A retrospective database analysis
Source: Influenza Other Respir Viruses. 2020 Jun 12;14(6):730–8. doi: 10.1111/irv.12770 (PMC7578296; doi:10.1111/irv.12770)
Supplement: Supplementary file 1 — Supplementary Material [file IRV-14-730-s001.docx]

**Supplementary Material**

**SUPPLEMENTARY TABLE S1** International Classification of Diseases (ICD) codes used to diagnose asthma wheezing

| **Pre-existing comorbidity** | **ICD-09** | **ICD-10** | **Procedure code** |
| --- | --- | --- | --- |
| Pre-term infants | 765, V21 | P07 | V2130, V2131, V2132, V2133, V2134, V2135 |
| Cerebrovascular disease | 430, 431, 432, 433, 434, 435, 436, 437, 438 | G45, G46, I60, I61, I62, I63, I65, I66, I67, I68, I69 | - |
| Ischemic heart disease | 410, 411, 412, 413, 414, 415, 416, 417, 420, 421, 422, 423, 424, 425 | A18, I20, I21, I22, I24, I25, I26, I27, I28, I30, I31, I32, I33, I34, I35, I36, I37, I38, I39, I40, I41, I42, I43, M32, T80, T81, T82 | - |
| Peripheral vascular disease | 443 | I73 | - |
| Down’s syndrome with CHD | 758 | Q90 | - |
| High-risk CHD | 425, 428, 745, 746, 747 | I42, I50, Q20, Q21, Q22, Q23, Q24, Q25, Q26 | - |
| Low-risk CHD | 745, 746, 747 | P29, Q20, Q21, Q22, Q23, Q24, Q25 | - |
| Neuromuscular disease | 330, 335, 343, 356, 358, 359 | E75, F84, G12, G31, G60, G71, G73, G80, G93 | - |
| Cystic fibrosis | 277 | E84 | - |
| Congenital and metabolic | 271, 272, 277, 740, 741, 742, 754, 756, 758, 759 | E71, E74, E75, E76, E77, E78, G90, Q00, Q01, Q02, Q03, Q04, Q05, Q06, Q07, Q67, Q76, Q79, Q87, Q89, Q91, Q92, Q93, Q95, Q96, Q97, Q98, Q99 | - |
| Kidney disease | 580, 581, 582, 583, 584, 585, 586, 587, 588, 589 | B52, E08, E09, M32, M35, N00, N01, N02, N03, N04, N05, N06, N07, N08, N14, N15, N16, N17, N18, N19, N25, N26, N27 | - |
| Asthma | 493 | J44, J45 | - |
| BPD | 770 | P27 | - |
| Influenza and pneumonia | 480, 481, 482, 484, 485, 486, 487, 488, 507, 514, 516, 770 | A22, A37, A48, B25, B44, B77, J09, J10, J11, J12, J13, J14, J15, J17, J18, J69, J81, J84, P23 | - |
| Chronic perinatal respiratory disease | 770 | P27 | - |
| Congenital airway anomalies | 748 | Q30, Q31, Q32, Q33, Q34 | - |
| Interstitial pulmonary fibrosis of prematurity | 770 | P27 | - |

BPD = bronchopulmonary dysplasia; CHD = congenital heart disease.

**SUPPLEMENTARY TABLE S2** Demographic characteristics of patients with 3 and 5 years of follow-up

|  | **3-year follow-up** | | | **5-year follow-up** | | | |
| --- | --- | --- | --- | --- | --- | --- | --- |
| **Patients, N (%)** | **All patients**  **N = 5902** | **High-risk-factor negative**  **n = 4524**  **(76.7%)** | **High-risk-factor positive**  **n = 1378**  **(23.3%)** | **All patients**  **N = 2340** | **High-risk-factor negative**  **n = 1788**  **(76.4%)** | **High-risk-factor positive**  **n = 552**  **(23.6%)** |  |
| Gender  Male, n (%) | 3366 (57.0) | 2551 (56.4) | 815 (59.1) | 1337 (57.1) | 1001 (56.0) | 336 (60.9) |  |
| Age  Mean (SD)  Median (IQR) | 0.8 (0.9)  0 (0–2) | 0.8 (0.9)  0 (0–2) | 0.8 (0.9)  0 (0–2) | 0.9 (0.9)  1 (0–2) | 0.9 (0.9)  1 (0–2) | 0.9 (0.9)  1 (0–2) |  |
| Race  African American, n (%)  Asian, n (%)  Caucasians, n (%)  Other/unknown, n (%) | 274 (4.6)  210 (3.6)  3976 (67.4)  1442 (24.4) | 219 (4.8)  158 (3.5)  3014 (66.6)  1133 (25.0) | 55 (4.0)  52 (3.8)  962 (69.8)  309 (22.4) | 99 (4.2)  87 (3.7)  1527 (65.3)  627 (26.8) | 83 (4.6)  64 (3.6)  1156 (64.7)  485 (27.1) | 16 (2.9)  23 (4.2)  371 (67.2)  142 (25.7) |  |
| Hospitalized with RSV, N (%) | 466 (7.9) | 313 (6.9) | 153 (11.1) | 168 (7.2) | 104 (5.8) | 64 (11.6) |  |

IQR = interquartile range; RSV = respiratory syncytial virus; SD = standard deviation.

**SUPPLEMENTARY FIGURE S1** Attrition table for analytical cohort selection

**
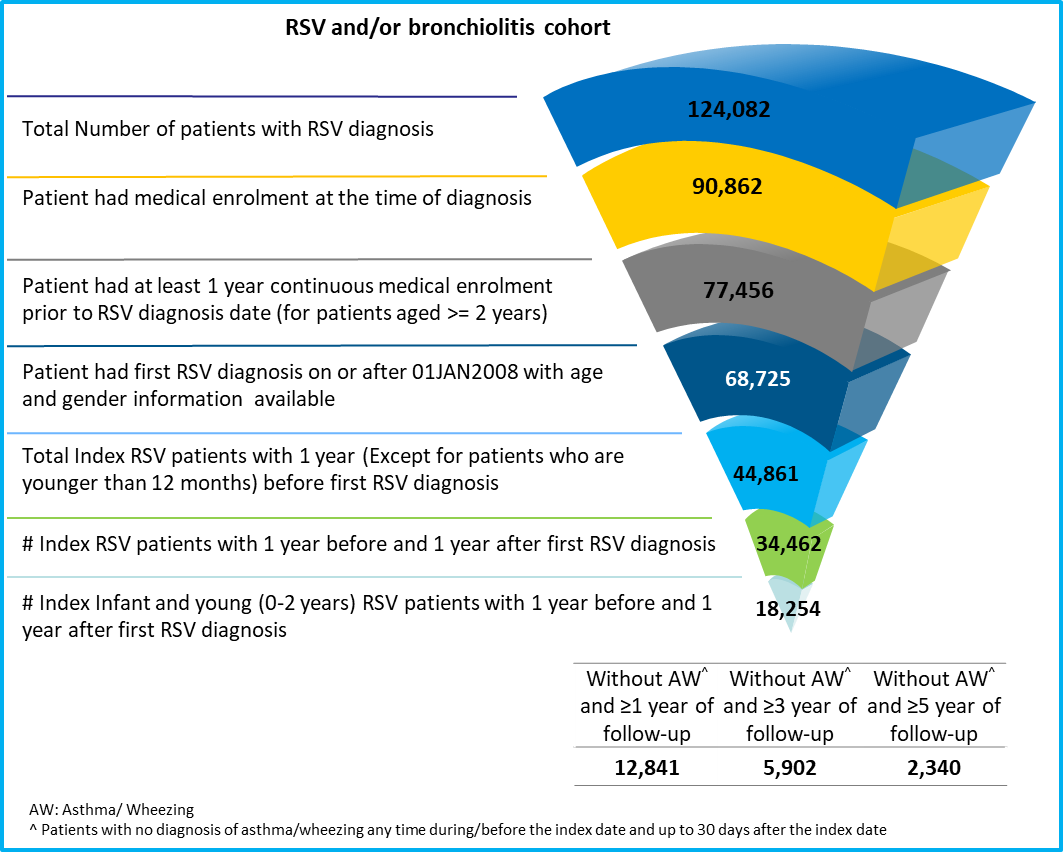
**
